# Production and Functionalities of Specialized Metabolites from Different Organic Sources

**DOI:** 10.3390/metabo12060534

**Published:** 2022-06-10

**Authors:** Abiodun Oladipo, Victor Enwemiwe, Onome Ejeromedoghene, Ademola Adebayo, Olakunle Ogunyemi, Fangfang Fu

**Affiliations:** 1 Co-Innovation Center for Sustainable Forestry in Southern China, College of Forestry, Nanjing Forestry University, Nanjing 210037, China; oladipoabiodun@njfu.edu.cn; 2 Department of Animal and Environmental Biology, Delta State University, Abraka 330106, Nigeria; enwemiwevictor@gmail.com; 3 School of Chemistry and Chemical Engineering, Southeast University, Jiangning District, Nanjing 211189, China; oejeromedoghene@seu.edu.cn; 4 Department of Forest and Conservation Sciences, Faculty of Forestry, University of British Columbia, Vancouver, BC V6T1Z4, Canada; aadebayo@alumni.ubc.ca; 5 Department of Social and Environmental Forestry, University of Ibadan, Ibadan 200005, Oyo State, Nigeria; olakunle4impact@yahoo.com

**Keywords:** bioactive compounds, medicinal plants, microbial benefits, plant-metabolite relationship

## Abstract

Medicinal plants are rich sources of specialized metabolites that are of great importance to plants, animals, and humans. The usefulness of active biological compounds cuts across different fields, such as agriculture, forestry, food processing and packaging, biofuels, biocatalysts, and environmental remediation. In recent years, research has shifted toward the use of microbes, especially endophytes (bacteria, fungi, and viruses), and the combination of these organisms with other alternatives to optimize the production and regulation of these compounds. This review reinforces the production of specialized metabolites, especially by plants and microorganisms, and the effectiveness of microorganisms in increasing the production/concentration of these compounds in plants. The study also highlights the functions of these compounds in plants and their applications in various fields. New research areas that should be explored to produce and regulate these compounds, especially in plants and microbes, have been identified. Methods involving molecular studies are yet to be fully explored, and next-generation sequencing possesses an interesting and reliable approach.

## 1. Introduction

Most plants exhibit a relationship with different microorganisms that can be beneficial or detrimental to their growth, development and/or survival. A clear understanding of these microbes and their ability to influence plant survival is of great importance. This is because the benefits derived from plants and their associated endophytes are numerous, and human survival depends largely on this. Endophytes consist of bacteria, fungi, and viruses that are mutualistic in nature, living inside plants where they spend part or all of their life cycles without causing harm to the host plant [1,2]. Generally, endophytes play an important role in suppressing biotic and abiotic stresses and activating defense mechanisms in plants, thereby contributing to growth and development [2,3]. Due to their unique nature, endophytes have been isolated from different plant parts, such as leaves [4], stems [5], roots [5], flowers [6], seeds [7] and fruits [8]. Different metabolites are found in living organisms, with a greater percentage found in plants; however, microbes are also a peculiar source of over 20,000 compounds that biologically and actively interfere with plant’s behavior and the survival of other living organisms [9]. The diversity of specialized metabolites encompasses groups of organic compounds, and they can be found in plants or produced by microorganisms, such as bacteria or fungi. These compounds may not necessarily contribute directly to the growth of plants, but they have been identified to improve the general health status of plants [10,11,12,13].

**Figure 1:**
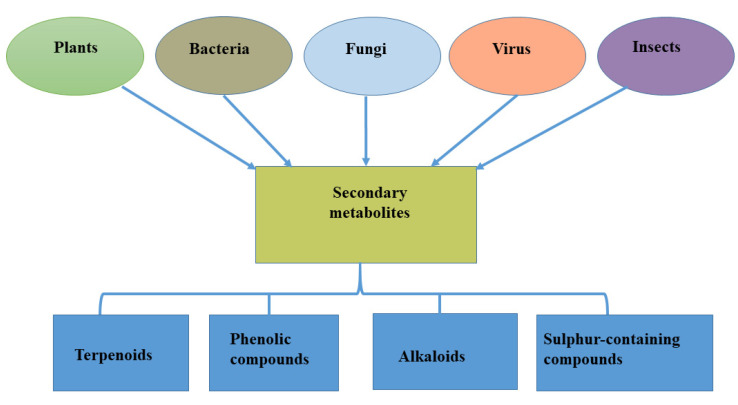
Sources of specialized metabolites and their major classifications.

Specialized metabolites may therefore be defined as natural products that are not mandated for vegetative growth of the host (plants) that produce them but could contribute to certain activities such as protection, molecular signaling, and environmental interaction of such a host, especially under challenging environmental conditions [14]. They are often found in small amounts in plant products and act as additional supplements [15]. More than 2 million specialized metabolites have been identified and grouped based on their function, structure, and biosynthesis. These compounds have been classified into four major groups: terpenoids, phenolic compounds, alkaloids, and sulfur-containing compounds [16] (Figure 1). Although produced in living cells, specialized metabolites contribute less to plant growth because of their low concentration [17].

Medicinal plants are known to house endophytes and their associated specialized metabolites, and due to the health benefits of these compounds, medicinal plants are largely exploited [18]. This vast diversity of natural resources (plants and associated endophytes) is a rich source of new biologically active molecules [19]. Several endophytic bacteria have been isolated from plants; in fact, all plants are known to house a variety of endophytes. Some of the common genera of endophytic bacteria that have been isolated from plants include *Bacillus* [20,21,22,23,24], *Pantoea* [22,23], *Streptomyces* [25] and *Enterobacter* [26]. However, a greater percentage of biologically active compounds isolated from bacteria vary, with the genus *Streptomyces* being the most examined for the production of specialized metabolites [13]. *Similarly*, a variety of endophytic fungi have been reported; common genera, among others, include *Fusarium*, *Colletotrichum* [27,28,29] and *Phoma* [29,30]. This review aims to provide dynamic information about specialized metabolites from organisms. In addition, it considers the production of specialized metabolites by (medicinal) plants and their associated endophytes, as well as the functions of specialized metabolites in these plants. It further examines the production of specialized metabolites by endophytic bacteria and fungi, the effect of viruses on their production in plants, and communicates more prospects in this area for novel findings and its application in different fields.

## 2. Plants as a Source of Specialized Metabolites and the Effect of Specialized Metabolites in Plants

Medicinal plants have been exploited for the extraction of bioactive compounds for a very long time. However, due to the difficulty that exists, especially with certain plants from specific biotopes and the challenges associated with field cultivation (susceptibility to pathogens), etc., researchers have explored other alternatives, such as tissue and organ culture, and plant cells, to produce these active compounds [10].

**Figure 2:**
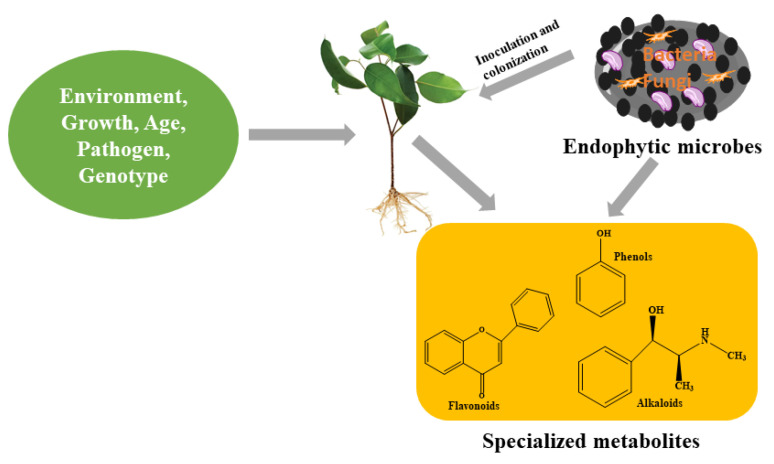
Endophytes and other factors influencing the production of specialized metabolites in plants.

Although the major function of specialized metabolites in a plant’s primary metabolism remains unclear [13], however, by employing molecular techniques, the importance of specialized metabolites to a plant’s general wellbeing has been identified. The interaction between plants and specialized metabolites contributes to the fitness of plants needed to withstand challenging environments. This is because these compounds, which they produce, perform an array of responsibilities at the cellular level to ensure the survival of plants. The production of specialized metabolites by plant cells are therefore actions taken by plants as they respond to challenges arising from biotic and abiotic stresses [10,31]. Basically, their function in physiological processes has been identified in response to stress and defense signaling, which results in their production. However, to understand the types and the level of production of specialized metabolites in plants, the consideration of certain factors such as genotype, species, environmental factors, age (Figure 2) and physiology cannot be overruled or jettisoned [32]. These factors, among others, present a holistic approach to better understanding the production of specialized metabolites.

Ibrahim et al. [33] in an experiment to understand the effect of genotype on the production of specialized metabolites, examined two different genotypes (salt tolerant and salt sensitive) of cotton under greenhouse conditions. It was observed that the flavonoid and phenol contents were significantly enhanced in salt-tolerant plants subjected to drought and drought plus salinity stress, while the phenol content was greatly reduced in salt-sensitive plants [33]. As observed by Ramírez-Briones, et al. [34], specialized metabolites produced in deciduous and perennial *Diospyros* species also varied, where total phenolic acids were observed to be higher in the leaves of *D. rekoi* compared to *D. digyna* during spring. This further reveals that variations in climatic and soil conditions, among other factors (genetics and biotic), could affect the production of bioactive compounds even among certain plants of the same genus [34].

Plants detect stress (biotic or abiotic) through signal responses and crosstalk, which, when activated, may trigger the production of specialized metabolites. The production of specialized metabolites under these conditions is remarkably one of the ways the immune system has evolved, thereby developing the ability to withstand such stress(es) [32]. This regulation pathway employs physiological and biological processes that lead to the adjustment of osmotic pressure of plant cells, prevent growth and/or infection arising from pathogenic microbes, and prevent oxidation of cell components [35,36], thereby strengthening the plant defense system against any form of stress. Specialized metabolites can be extracted from any plant part; nevertheless, the location where biosynthesis occurs is confined to certain organs in most instances. It finds its way to other parts of the plant, where it can be stored (vacuole) using apoplast, symplast, or vascular tissues (which allows for movement of low molecular weight substances) as a channel of movement [37,38]. These bioactive compounds could be stored in plant tissues, such as roots, stems, leaves, leaves, somatic embryos, callus, or even flowers [32]. However, in plants, several biosynthetic pathways, such as the movalonic acid pathway, acetate–malonate pathway, 2-C-methyl-D-erythritol-4-phosphate pathway, etc., are linked to the production of these compounds [39].

## 3. Production of Specialized Metabolites by Plant’s Endophytes

The production of novel biological compounds by endophytes has gained the attention of researchers because, through careful examination, biologically active specialized metabolites have been reported to show activity against pathogens [40]. This is because they possess unique properties, such as antiviral, antibacterial, and anticancer properties; hence, they may be termed biologically active antimicrobial natural products [41,42]. Endophytes are known to be associated with different plant parts (roots, stems, leaves, and other tissues), providing an array of support to plants, ranging from nutrient uptake to defense against biotic stress [42] and tolerance to harsh environmental conditions [43,44,45]. Apart from these benefits, endophytes isolated from different plants or specifically different plant tissues have expressed the ability to produce specialized metabolites; these endophytes could be bacteria [46] or fungi [47] while viral infection also contributes to the production of specialized metabolites [48] (Figure 1).

## 4. Bacteria

In an experiment, four strains of endophytic bacteria belonging to *Chitinophaga* sp., *Allorhizobium* sp., *Duganella* sp., and *Micromonospora* sp. isolated from the roots of the *Alkanna tinctoria* were reported to have significantly increased alkannin and shikonin in the hairy roots of inoculated plants when compared to the uninoculated control. Considering the results of this experiment, the possibility of combining endophytes with the potential for plant growth promotion and enhancement of specialized metabolites as a means to generate increased production of specialized metabolites in selected medicinal plants cannot be overruled [46]. Bacteria isolated from grassland soil have also expressed the ability to synthesize specialized metabolites genetically. Crits-Christoph et al. [49] genetically identified that these organisms encode different polyketide and non-ribosomal peptide biosynthetic gene clusters, and further revealed that these microorganisms are members of the *Acidobacteria*, *Verrucomicobia*, and *Gemmatimonadetes*, and the candidate phylum *Rokubacteria* [49]. The most abundant bacterial phylum observed in soil biomes (*Acidobacteria*) according to Fierer, N. [50] actually expressed large numbers of biosynthetic genes; it was inferred that these gene clusters could synthesize non-ribosomal peptides (NRPs), polyketides, terpenes, bacteriocins, lassopeptides, lantipeptides, and metabolites of uncertain function. In total, 240 non-ribosomal peptide synthases (NRPSs), polyketide synthases (PKSs), and hybrid (NRPS-PKS) gene clusters were confirmed on the contigs from the four phyla [50].

In an attempt to identify endophytic bacteria and their biological compounds, 13 isolates from the leaves of *Anredera cordifolia* were examined [51]. These isolates, which belong to *Pseudomonas* sp., particularly *Pseudomonas aeruginosa*, produced specialized metabolites that expressed effectiveness against bacteria and showed antioxidant activities. *Pseudomonas* sp. has been identified as a major endophytic bacteria frequently isolated from medicinal plants [52,53,54]. The biological compounds produced by *P. aeruginosa*, as identified by Nxumalo and colleagues, included xanthoxylin, trans-2-decenoic acid, [1,2,4] oxadiazole, 5-benzyl-(thiophen-2-yl), dodecenoic acid, 3-nonynoic, pyrrolo [1,2-a] pyrazine-1,4-dione, hexahydro-3-(2-methylpropyl), pentadecanoic acid, and diisooctyl phthalate, which are recognized for antimicrobial activity while cis-9-Octadecenoic acid, 2-dodecenoic acid and 9-octadecenamide possess antioxidant properties [51]. Other bacteria species that have been identified as contributing to specialized metabolites in plants are *Bacillus amyloliquefaciens* [55] and *Bacillus cereus* [56] (Table 1).

Whole genome sequencing of microorganisms revealed the possibility of bacteria [66] to produce biologically active compounds. Whole genome sequencing of *Streptomyces avermitilis* ATCC 31267, which produces avermectin, showed 24 gene clusters for specialized metabolites, siderophores, and spore pigments whose structure and function have yet to be determined [66].

## 5. Fungi

The production of specialized metabolites by endophytic fungi and the effect of these fungi on the concentration of specialized metabolites produced by plants have also been studied. Four endophytic fungi, *Talaromyces* sp. and *Schizophyllum* sp., with the remaining two belonging to *Aspergillus* sp. isolated from the fresh root of the medicinal plant *Vernonia anthelmintica*, were examined for biological activities by Rustamova and colleagues [67]. The ethyl acetate extracts of these fungi expressed antimicrobial, cytotoxic, and antioxidant, antidiabetic, melanin content, and tyrosinase activity on murine B16 cells. The ethyl acetate extracts from these fungi have been suggested to contain compounds such as steroids, terpenes, or terpenoids [67]. The antibacterial activities of 24ξhydroperoxy-24-vinyllathosterol which is a steroid isolated from *V. anthelmintica,* were reported to be antibacterial against *B. cereus*, *S. aureus*, *B. subtilis*, and *E. coli*, with minimum inhibition concentrations ranging from 3.15 to 15.5 µg/mL [68].

Cryptotanshinone, which has been used in the treatment of several ailment [69,70,71], is the main bioactive compound of *Salvia abrotanoides* [72] and has been reported to be a product of endophytic fungi. To understand the discrepancies that may exist in the endophytic fungi of native *S. abrotanoides*, *S. abrotanoides* from three different geographical locations in Iran were examined. A total of 56 isolates were identified, and *Penicillium canescens*, *P. murcianum*, *Paraphoma radicina*, and *Coniolariella hispanica* (isolates) were able to produce cryptotanshinone independently, as reported by Teimoori-Boghsani et al. [73]. Furthermore, a variety of compounds, such as terpenes, isoflavons, ketons, phenols, lipids, alkaloids, and polyketides, were said to have been produced by fungi (Table 1) belonging to *Penicillium*, *Talaromyces*, *Fusarium*, *Paraphoma*, and *Coniolariella* genera. However, the increase in the production of cryptotanshinone in *S*. *abrotanoides* was due to the exogenous gibberellin and endophytic fungi. Also, fungi cultivated under laboratory conditions also yielded a substantial amount of cryptotanshinone when supplemented with gibberellin [73].

Plants subjected to stress have expressed variations in the composition of endophytic fungi present in them; such variations were expressed in an experiment conducted by Mefteh and colleagues [74] by isolating and identifying 52 fungi from healthy and brittle leaf-diseased date palm trees (*Phoenix dactylifera* L.). However, 44.7% of the fungi isolated were specifically from brittle leaf-diseased date palm trees. More interestingly, the ethyl acetate extract of two isolates, *Geotrichum candidum* and *Thielaviopsis punctulate*, exhibited antibacterial activity against pathogenic bacteria *Micrococcus luteus* and *Bacillus subtilis*, while isocumarin and triterpenoids were extracted as biological compounds from *Geotrichum* sp. [74]. This confirms that plants affected by stress do not only possess hidden resources but could actually be key to novel and reliable sources of bioactive compounds.

Single strains or species of fungi exhibit a vast diversity of metabolites. Certain classes of fungi, such as Basidiomycetes, are underexploited [75], and by thorough examination of different genera, it is possible to identify several novel species with unique applicability, especially in the drug and pharmaceutical industries. Some fungi that may have been neglected as reported by Leman-Loubière et al. [76] could be studied more extensively, as it appears that they hold a wide array of biological compounds; for example, *Daldinia eschscholtzii*, *Hypoxylon rickii*, and *Pestalotiopsis fici* have been reported to produce metabolites of great importance [77]. *Aspergillus ochraceus* DSM 7428, which is known to produce only aspinonene as a major compound, was found to produce 15 additional compounds when the mechanism of “one strain many compounds” was explored, resulting from different biosynthetic pathways [78,79]. The production of these additional compounds was attributed to the variation in culture conditions and the analysis of the culture broth in details [79]. Additionally, novel polyketides and sordarin derivatives, which were produced by *Xylotumulus gibbosporus* belonging to the small genera of *Xylariaceae* [80,81] further proves that some fungi have not been fully exploited for specialized metabolites.

Mycorrhizal fungi are known for their symbiotic relationship with plants. These types of fungi have been reported to significantly influence specialized metabolites in plants. In an experiment, Pistelli and colleagues [82] investigated how arbuscular mycorrhizal fungi (AMF) affect phytochemicals in *Bituminaria bituminosa*. The medicinal plant *B. bituminosa* was inoculated with arbuscular mycorrhizal fungi, and the phytochemicals were examined during the vegetative and flowering stages. It was observed that although plants inoculated with arbuscular mycorrhizal fungi reduced the production of pterocarpans (bitucarpin A and erybraidin C) during the flowering stage, plants inoculated with arbuscular mycorrhizal fungi exhibited higher amounts of furanocoumarins and pterocarpans during the vegetative stage when compared to non-inoculated plants where these compounds were not detected at all [82].

Arbuscular mycorrhiza fungi belong to a group of soil-dwelling microbes with an active role in the mediation of secondary metabolism and the production of biologically active ingredients in medicinal plants [83]. Eight different species of AMF (*Glomus formosanum*, *Glomus tenebrosum*, *Septoglomus constrictum*, *Funneliformis geosporum*, *Rhizophagus manihotis*, *Ambispora gerdemanii*, *Acaulospora laevis* and *Acaulospora tuberculate*) were isolated from soil samples of *Salvia miltiorrhiza* and their effect on the growth of *S. miltiorrhiza* under greenhouse condition along with the production of specialized metabolites were examined by Wu and colleagues [83]. Aside from the fact that AMF generally increased the root biomass of *S. miltiorrhiza*, it was also observed that inoculation of *S. miltiorrhiza* with the abovementioned AMF had great influence on phenolic acids when compared to tanshinones; in particular, *G. formosanum*, *A. gerdemanii*, and *A. laevis* significantly increased phenolic acid, while inoculation with both *F. geosporum* and *A. laevis* significantly reduced total phenolic acids [83]. Overall, native AMF increased production of specialized metabolites and plant’s biomass as a result of better nutrition, while the involvement of genes responsible for plant defense mechanisms increased phenolics in medicinal plants, as presented by Wu et al. [83].

**Figure 3:**
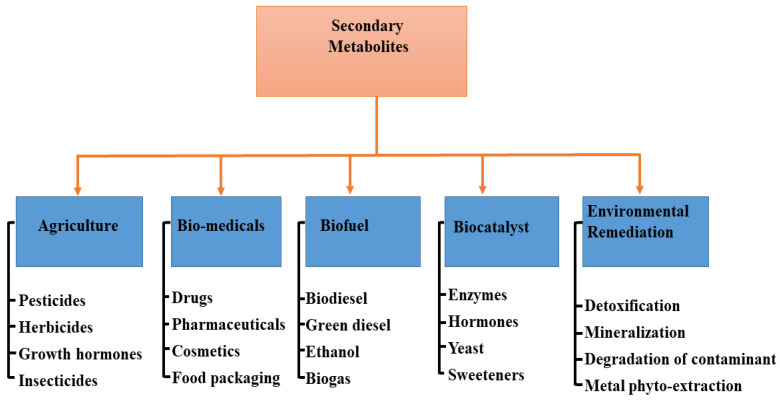
Application of specialized metabolites in different fields.

In different industries, the application of specialized metabolites varies (Figure 3). For instance, in agriculture, the nutritional value of edible vegetables could be increased, resulting in health improvement when consumed, although food intake may not necessarily be increased [84]. In most cases, the nutritional values of such vegetables are associated with the biological compounds present in them. Mycorrhizal fungi are therefore essential in the assembling and storage of bioactive compounds and, as such, are valid alternatives to chemical fertilizer in sustainable agriculture [85]. However, the mechanism employed by AMF in influencing the production of specialized metabolites is yet to be made clear [86], though some have attributed it to the vegetative response to colonization [87] and an increase in the activities of enzymes [88].

## 6. Effect of Viruses on Plant’s Specialized Metabolites

Plant viruses are generally believed to be detrimental to the growth, development, and survival of plants. There are many reported cases of viral infection, especially in agricultural crops. For instance, *Tobacco mosaic virus*, which is in fact the first virus to be identified, is responsible for mottled browning of tobacco leaves, and it spreads mechanically by accessing open surfaces or injured plant parts. Notably, this virus does not only affects tobacco but has also been reported to affect tomato as well, with the best solution being the destruction of infected plants [89]. Other plant viruses that have been identified to be detrimental to plants include *Tomato spotted wilt virus,* which accounts for a loss of more than one billion United States dollars in 1994 [90], *Potato virus X*, *Tomato yellow leaf curl virus* transmitted by whitefly, which also causes tremendous economic loss, *Cauliflower mosaic virus*, and *Plum pox virus* [91].

Although there are few reports relating to viruses and forest tree species, especially coniferous trees, some viruses that have been identified are associated with the death of tree species. Scots pine mosaic and scots pine bushy stunt virus were identified in *Pinus sylvestris* [92,93] and according to Biddle and Tinsley TW [94] similar virus diseases were identified in *Pinus monticola* in Great Britain. Other viruses associated with damage in tree species include *Isometric labile ringspot viruses*, *Alfalfa mosaic virus*, *Prunus necrotic ringspot virus*, *Prune dwarf virus*, *Apple mosaic virus*, and *American plum line pattern virus* [95]. However, some viruses have been reported to be beneficial to plants, especially agricultural crops. The beneficial effect of the virus *Cucumber mosaic virus* (CMV) significantly enhanced drought and freezing tolerance in beet plants when compared with uninoculated plants [96]. In *Arabidopsis thaliana*, the 2b protein of CMV increased drought tolerance, accounting for 40% difference in water loss between control and transgenic plants [97]. Mycoviruses are associated with fungi and, as such, have proved helpful, especially with high temperature. An example was reported by Márquez et al. [98], where the *Curvularia thermal tolerance virus* contributed significantly to high temperature (65 °C) tolerance in the association between the endophytic fungus *Curvularia protuberate* and *Dichanthelium lanuginosum* (panic grass). Apart from these, viruses could also influence plant’s nodulation, especially in the presence of nitrogen, thereby affecting the overall growth and development of plant [99].

Although viruses may appear harmful to plants, it is clear that they can also contribute to abiotic stress tolerance in plants; hence, there is the possibility of increasing the production of specialized metabolites. Lan and colleagues [62] observed that although CMV adversely affected the physical properties (fruit height, fruit width, and fruit weight) of *Passifolia edulis*, the fruit and leaves of infected *P. edulis* showed increased polyphenolic and flavonoids. The results reported that CMV accounted for an increase of 28.7% and 26.1% in the total polyphenol contents of the fruit and leaves of *P. edulis*, respectively, while the same virus accounted for an increase of 58.3% and 48.1% in the flavonoid contents of the same tissue, respectively [62].

Camalexin, a bioactive compound that is essential in the defense of plants against pathogenic attack, was induced in *Arabidopsis thaliana* as a result of the virus [100,101]. Likewise, the production of hydroxycinnamic acids and flavonols consisting of kaempferol, quercetin derivatives, and myricetin was enhanced significantly by an RNA virus, notably in *Grapevine leaf roll-associated virus 3* in the *Vitis vinifera* white cultivar Malvasía de Banyalbufar [102,103]. As reported by many authors, viruses do not have their own metabolism; therefore, the production of metabolites by virus strains only appears impossible, as this has not been reported because they are arguably living or non-living entities; however, they possess genes associated with living cells. They depend solely on the metabolic mechanism of their host, and through systemic manipulation, they find their way into plants and replicate, causing infection in such plants [104,105]. Therefore, the possibility that a virus adversely affecting a particular plant could be beneficial to another plant species cannot be overruled (Table 1); however, this depends on certain factors, including plant genotype and environmental conditions. However, exploring forest tree species as they relate to viral infection has not been critically examined; as a matter of fact, there are still unknown or yet to resolved margins in plant–virus–environment interaction.

## 7. Insects and Specialized Metabolite Production

Insects and plants have a long history of coexistence, as well as endophytes and plants. As a member of invertebrates, insects occupy more than three-quarters of the earth, playing significant roles in ecosystem functioning, including predation, herbivory, parasitism, pollination, detritivory, and so on. Insects, microorganisms, and plants interact in a complex way, which forms an integral part of the ecosystem and links entomology to pathology. Studies involving insect association with endophytes, such as bacteria and fungi, are evolving, predicting their potential in industry, agriculture, medicine, and many other areas [106,107]. The presence of endophytes, as well as associating insects, act as foreign bodies to the plant that release hormones, causing changes in the plant’s activities and thus leading to the biosynthesis of vital pesticidal agents against diseases [108].

In plants, the association of insects and endophytes do not only affect the overall biomass, nitrogen supplementation, and food supply in the ecosystem, but also acts as a barrier to plant–herbivorous insect competition. Metabolic products of endophytes and pathogenic fungi, bacteria, and viruses from insects have been used as biocontrol agents against the pest activities of insects [109,110]. It has been reported that the adoption of biologically-based control involving insect pathogenic microbes was successful and effective against a broad spectrum of insect pests both in storage and field condition [111,112,113].

Specialized metabolites have also been produced by insects, as well as microbes isolated from insects [114]. Some of these insects have also been reported to influence the production of bioactive compounds in plants, with damage ranging from mild to severe [115]. As experienced in plants, insects also employ specialized metabolites for defense. For example, the frontal glands of advanced termite soldiers could release terpenes as counteractions against attackers [116]. Additionally, as a strategy of attraction, monoterpenes and sesquiterpenes play an important role in communication to attract mates and track food sources [117,118]. In line with insect infestation, the relationship between plants, insects, and metabolites was partly examined by Koch et al. [119] and showed that yellow sugarcane aphids significantly contributed to salicylic acid levels and enriched flavonoids in *Panicum virgatum*.

An invasive insect prominent in North America, Hemlock woolly adelgid, has been reported to increase the attraction of folivorous insects to hemlock [120]. However, in an experiment to examine the infestation of this insect on the jasmonic acid of *Tsuga canadensis*, Rigsby and others [120] observed that Hemlock woolly adelgid contributed to the systemic response of the plant. Furthermore, when Saad and colleagues [121] investigated the effect of previous infestation of *Capsicum annuum* plants by green peach aphid (*Myzus persicae*) on the olfactory behavioral response of *Bemisia tabaci*, they reported that female *B. tabaci* preferred non-infested plants to pre-infested plants; this suggests that pre-infested plants may contain compounds that prevent or reduce infestation by this insect. Additionally, it was observed that plants infested by green peach aphid significantly increase in the production of monoterpenes (cymene; 1,8-cineole), sesquiterpenes (β-cadinene, α-copaene), and methyl salicylate (MeSA) compared to non-infested plants, which further suggests that plants infested by green peach aphid might be capable of inducing the production of specialized metabolites that deter *B. tabaci* from settling on its host plants [121].

## 8. Application of Specialized Metabolites from Organic Sources

The specialized metabolites isolated from endophytic microbes and plants have been characterized by numerous bioactive compounds or phytochemicals (Table 1), which broaden the scope of their application across many fields of scientific research.

## 9. Agriculture (Agrochemicals)

The agricultural sector is one of the fastest-growing sectors in the world economy due to the rise in global population and the increase in food production. Endophytes are known to produce a range of metabolites useful in agriculture for growth regulators and pesticides (Figure 3) that are applicable to several economically important plants [122]. In modern-day intensive farming technology, specialized metabolites produced by agriculturally important microorganisms have been explored in many ways to improve the quality of crops [123]. This is achievable owing to the fact that these plant–microbe could serve as green alternatives toward producing materials for combating biotic and abiotic stressors, and offer promising sources of new biorational compounds [124]. For example, *Actinobacteria* and *Bacillus* endophytes produce aromatic compounds, lipopeptides, plant hormones, polysaccharides, and several enzymes linked to phenylpropanoid metabolism, thus representing a high potential for promoting plant growth and crop disease management [44]. Moreover, some endophytes containing antibiotics located in the rhizosphere can be employed to control the growth of harmful bacteria [125]. Furthermore, the endophytic microbes can also mediate plant adaptation to environmental stress due to conditions such as temperature, drought, cold stress, heavy metal accumulation, and high-energy ultraviolet radiation with a wavelength of around 280–315 nm [126,127].

## 10. Biomedical Application

Endophytes are known to produce a diverse range of natural products with numerous biomedical functions, such as pharmaceuticals, drug delivery agents, cosmetics, and food packaging/preservative materials (Figure 3). This is due to the presence of bioactive compounds, including alkaloids, flavonoids, terpenes, steroids, curcumins, saponins, and phenolics, all of which can potentially suppress bacteria and fungi pathogens and curb new emerging infectious diseases [128,129,130]. The isolation and identification of *Methylobacterium radiotolerans* MAMP 4754 from the seeds of *Combretum erythrophyllum* was investigated and confirmed with high antimicrobial and antioxidant activity, which is associated with the production of plant-derived specialized metabolites by this strain [131]. In addition, mangrove fungal endophytes have been evaluated against a panel of human pathogenic microbes and cancer cell lines because they can produce an impressive panoply of metabolites with promising biological activities [132]. Moreover, in the field of nanomedicine, specialized metabolites from microbes have been classified as apt to absorb and accrue metal ions. They can also serve as nontoxic and ecofriendly reducing agents to control the topology and morphology of nanomaterials with tunable properties that can be used as chemotherapeutic, and antibiotic agents [133,134]. For example, Munawer and others [135] prepared gold nanoparticles (AuNPs) from the aqueous extract of the endophytic *Cladosporium* sp. (MycoAuNPs) isolated from *Commiphora wightii*, with promising anti-breast cancer activity in the MCF-7 cancer cell line. In another study, the endophytic bacterial strain *Rothia endophytica* isolated from healthy maize roots was used to synthesize silver nanoparticles (Ag-NPs). The cubic-shaped Ag-NPs obtained displayed improved anti-candidal activity with minimum inhibitory concentration (MIC) and minimum bactericidal concentration (MBC) at 62.5 and 125 µg/mL, respectively [136]. Neethu and colleagues [137] biosynthesized Ag-NPs from the marine endophytic fungus *Penicillium polonicum* with MIC and MBC efficacy of 15.62 and 31.24 µg/mL respectively against biofilm forming, multi-drug resistant *Acinetobacter baumanii*. The aqueous fungal extract of *Periconium* sp. was deployed as a chelating agent for the Zn^2+^ ions for the biosynthesis of ZnO nanoparticles with highly improved antibacterial and antioxidant properties [138].

## 11. Biofuel

Microalgae are very rich lipids and accumulate specific specialized metabolites, which are high-value products for the production of alternative fuels [139,140]. The lipid content of microalgae is ~20–50% of the cell dry weight, and <80% under certain conditions [141,142]. For instance, Rodolfi et al. [143] performed a screening process on 30 microalgal strains for biomass productivity and lipid content. The authors deduced an increase in both lipid content and areal lipid productivity attained through nutrient deprivation in an outdoor algal culture. This implies that the marine eustigmatophyte has the potential for an annual production of 20 tons of lipid per hectare in the Mediterranean climate and of more than 30 tons of lipid per hectare in sunny tropical areas. These species of organisms are among the fastest-growing plants and can serve as a sustainable energy source for the production of biodiesel (Figure 3) and several other biofuels by converting sunlight into chemical energy. Biofuels obtained from microalgae are renewable, nontoxic, biodegradable, and environment friendly [144]. The oil amassed in most microalgae is mainly triglyceride, which can be utilized for the production of biodiesel and glycerol via transesterification reaction [145]. Miao and Wu [146] presented an integrated approach for the production of biodiesel from microalgal oil based on the heterotrophic growth of *Chlorella protothecoides,* with an accumulated 55% lipid content in cells. The optimized transesterification process, which occurred in 4 h, produced 68% biodiesel with a specific gravity of 0.8637 at a 56:1 molar ratio of methanol to oil at 30 °C using sulfuric acid as the catalyst. However, Xiong and others [147] showed that the high-density fermentation of microalga *C. protothecoides* in bioreactors could produce up to 98% biodiesel production catalyzed by lipase. Moreover, Abou-Shanab and colleagues [148] utilized municipal wastewater for the culture of *Scenedesmus obliquus* and *Micractinium reisser,* with remarkable biomass yields (0.41 ± 0.01 and 0.26 ± 0.03 g dry wt. L^−1^) and lipid content (22% and 19%), respectively, which are desirable properties for biodiesel production. In other studies, Gao et al. [149] prepared biodiesel from the lipids of *C. protothecoides* through acid-catalyzed transesterification with the incorporation of sweet sorghum juice to enhance lipid production. Meanwhile, Lu et al. [150] used cassava hydrolysate as an alternative carbon source for the growth of microalgae (*C. protothecoides*), having about 53% lipid content for the optimized production of biodiesel with good fuel properties.

## 12. Biocatalysts

Generally, primary metabolites are enzymes, and they can be obtained from plants and microbes [151] (Figure 3) with broad bioactivity; however, they are essential in the synthesis of specialized metabolites [152]. Microbial enzymes have more advantages than enzymes derived from plants or animals because of the following characteristics: broad biochemical diversity, large culture quantities, easily manipulated genetically, more catalytic activity, reduced costs of production, equipment availability, and sustainability [153]. Owing to the wide production of flavonoids, terpenoids, and polyketide antibiotics, plant endophytes have emerged as new in vitro and in vivo biocatalytic materials for engineering glycosylation toward producing a large repertoire of versatile glycoprofiles [154]. Endophytic microbial isolates also find wide application as industrial catalysts toward the production of biofuels [147] and manifold biotransformation of exobiotic substrates, particularly in redox reactions [155,156]. For example, the endophytic yeast *Candida guillermondi* isolated from castor leaves (*Ricinus communis* L.) was optimized and characterized with promising properties as a biocatalyst for the synthesis of esters in the food and biofuel industry [157]. In addition, the immobilization of *Candida antarctica* enzyme onto a macroporous acrylic resin displayed intriguing biocatalytic performance toward the preparation of dichloropropyl acrylates from dichloropropyl dodecanoates via transesterification reactions [158]. In many industrial processes, these isolates have been utilized severally in the bioreduction of ketones, e.g., acetophenone [159], propiophenones [160] cocktail of ketones [161], etc. to alcohol under ambient conditions and increased product yield.

## 13. Environmental Remediation

The rapid increase in world population and high level of industrialization have engineered the continuous pollution of the environment from different sources and reduced life expectancy in different parts of the world. Consequently, specialized metabolites produced by endophytic microbes have been seriously exploited as avenues to promote a green environment as a potent bioremediation (Figure 3) tool for the adsorption, removal, detoxification, and degradation of many organic pollutants [162]. For instance, the endophytic bacterium *Methylobacterium extorquens* C1 isolated from ryegrass was used for the sorption and enzymatic degradation of polycyclic aromatic hydrocarbons (PAH), with a removal rate increased by ~18.3–35.0% [163]. In addition, inter-planting ryegrass with *Seduce alfredii* with regular re-inoculation with *Microbacterium* sp. KL5 and *Candida tropicalis* C10 in the co-contaminated soil showed remarkable PAH removal (96.4%), PAH mineralization, and metal phytoextraction (36.1% Cd and 12.7% Zn) in a greenhouse study [164]. The endophytic fungus *Phomopsis liquidambari* has been investigated widely for the degradation of the xenobiotic compound (sinapic acid) in contaminated industrial wastewater and soil [165]; and has been reported as a suitable material for mitigating (biodegradation) the allelopathic stress caused by cinnamic acid in continuous cropping soils [166]. In another approach, Fu et al. [167] identified that the combination of *P. liquidambari* with rice is very potent in the elimination of phenanthrene accumulated in vivo in rice seedlings, with a 25.68% increase in the removal rate in an inoculated treatment compared to the uninoculated treatment after cultivation for 30 days. In addition, slop oil from oil refining was found to be degraded and removed by endophytic *B. cereus* EN18 with biotransformation [168]. Furthermore, in the management of toxic metal contaminants, Jeyasundar, and others [169] studied the use of bacterial consortium and *Brassica juncea* to improve soil properties and enhanced phytoextraction of Cd, Cu, Pb, and Zn, heavy metals polluted mining soil. Bilal and colleagues [170] determined that Glycine max L (soybean) plants inoculated with *Sphingomonas* sp. LK11 are capable of reducing oxidative stress and the translocation of Chromium (IV) to the roots, shoot, and leaves of the plant, and also downregulate the synthesis of endogenous defense-related phytohormones. The interactive effect of *Enterobacter* sp. MN17 and biochar was also projected as an effective remediation strategy for Cd-contaminated soil for sustainable crop production [171]. Additionally, endophytic microbes have been identified as rich sources of metabolites that can be employed for the biogenic synthesis of nanostructured devices that can serve as bioreceptors and promise to be candidates for the efficient monitoring and treatment of emerging contaminants in the ecosystem [172].

## 14. Future Research Directions

Bioactive compounds have various applications (Figure 3) that are not only crucial to the survival of plants and animals but also to that of humans. Due to their large biological activities, plant-specialized metabolites have been used for centuries in traditional medicine. In plants, their functionalities include protection from biotic and abiotic stresses, enhancement of the symbiotic relationship between plants and other microbes, i.e., bacteria, fungi, and viruses), participation in hormonal regulation, and acting as agents of metal degradation and transportation. They are also well noted agents in antibacterial, antifungal, antiviral, allelopathic, anti-germination, and phytoalexin activities. As important as they are, certain factors also affect their production as well; hence, there is recent interest in the study of such factors both under in vitro and in vivo growth conditions. Although the duration of inoculation may also contribute to the production of specialized metabolites, the increase or decrease largely depends on the plant species, the interaction between plant and endophytes, genetic manipulations, and the influence of environmental and/or biotic stress on the plant. The presence of distinct microbes (bacteria, fungi, and viruses) communities in different plant species and among compartments of the same plant species could account for the differences in the medicinal properties of the two plants. It is worth noting that although an increase in the concentration of specialized metabolites in plants enhances plant’s defense mechanism, it could also lead to negative effects, such as slower growth rate or delayed reproduction in plants and in extreme conditions, death. Considering the changes in climatic conditions that have plagued the global ecosystem, specialized metabolites produced by plants and other organisms present one possible approach that can be employed by plants, especially to tolerate varying climatic conditions and resist biotic stresses. Therefore, the mechanisms that support both the production and regulation of bioactive compounds, particularly in plants and microbes, should be holistically examined.

Considering the broad field of nanomedicine, the preparation of nontoxic and ecofriendly nanostructured drug delivery agents and nanofertilizers with specialized metabolites from endophytic microbes is still in their infancy. The multi-biofunctionality of endophytic microbes can serve as a promising candidate for the slow release and target-specific delivery of bioactive compounds as well as fertilizer compounds that can promote the uptake of micro- and macronutrients needed for plant growth and provide defense support for the plant against environmental contaminants. These metabolites, which are fundamental to plants, animals, and human health systems, have become the building block employed by researchers to develop or formulate bioactive compounds into useful substances with a wide range of applicability in many fields. Exploring molecular biology has paved the way for more advanced research to further examine the relationship/correlation between phenotype, plant physiology, tissue culture, and unique genes that can be manipulated for the production and regulation of these compounds in plants to survive both biotic and abiotic challenges. Therefore, methods such as bioinformatics, phylogenomics, metabolomics, and transcriptomics should be well examined, employing next-generation sequencing to develop reliable methods for the production and regulation of specialized metabolites.

## Figures and Tables

**Table 1:** Percentage differences in the production of bioactive compounds as affected by microorganisms in plants.

S/N	Plants	Microorganisms	Bioactive Compounds	Concentration from Inoculated Plants (%)	Concentration from Non-Inoculated Plants (%)	Differences (%)	References
1	*Rumex gmelini*	*Aspergillus* sp. (fungus)	Resveratrol	83.33	16.67	66.66	[57]
			Chrysophaein	69.57	30.43	39.14	
			Musizin	0	100	100	
			Chrysophanol	76.19	23.81	52.38	
2	*Menth piperita*	*B. amyloliquefaciens* (bacteria)	Linalool	82.14	17.86	64.28	[55]
3	*Saliva miltiorrhiza*	*B. cereus* (bacteria)	Tanshinone	85.27	14.27	71.00	[58]
4	*Anoectochilus roxburghii*	*Chaetomium*	Isoquercitrin	60.81	39.18	21.63	[59]
		*globosum*	Narcissin	35.75	64.24	28.49	
		*Colletotrichum*	Rutin	63.45	36.55	26.90	
		*gloeosporioides*	Isoquercitrin	54.23	45.77	8.46	
		(fungi)	narcissin	64.64	35.35	29.29	
5	*Lycoris radiata*	*Phyllosticta ophiopogonis*	Narciclasine	57.14	42.86	14.28	[60]
		*Stagonosporopsis cucurbitacearum*	Lycorine	56.10	43.90	19.27	
			Galanthamine	62.75	37.25	25.50	
		*Phyllosticta capitalensis*	Lycoramine	58.54	41.46	17.08	
		*Glomerella magna* (fungi)	Tazettine	57.78	42.22	15.56	
6	*Fragaria x ananassa* var. Selv	*Pseudomonas* sp. (bacteria)	cyanindin 3-glucoside	73.08	26.08	46.16	[61]
7	*Passiflora edulis*	*Telosma mosaic virus*	Total phenolics	59.55	40.45	19.10	[62,63]
		*Cucumber mosaic virus* (viruses)	Total polyphenols	64.35	35.65	28.70	
8	*Pelargonium graveolens*	*Macrophomina pseudophaseolina* (fungus)	Citronellol	55.50	45.50	11.00	[64]
			geraniol	52.00	48.00	4.00	
9	*Andrographis paniculata*	*Macrophomina pseudophaseolina* (fungus)	Andrographolide	60.50	39.50	21.00	[64]
10	*Artemisia pallens*	*Fusarium redolens Phialemoniopsis cornearis* (fungi)	Davanone	56.95	43.05	13.90	[64]
			Ethyl cinnamate	55.50	45.50	11.00	
11	*Panax quinquefolius*	Fusarium solani	Ginsenoside-Rd	67.50	32.50	35.00	[65]
			Ginsenoside-Rc	78.00	22.00	56.00	
